# Mean signal and response time influences on multivoxel signals of contextual retrieval in the medial temporal lobe

**DOI:** 10.1002/brb3.302

**Published:** 2014-12-24

**Authors:** Emilie T Reas, James B Brewer

**Affiliations:** 1 Department of Neurosciences, University of California, San Diego9500 Gilman Dr., La Jolla, California, 92093-0949; 2 Department of Radiology, University of California, San Diego9500 Gilman Dr., La Jolla, California, 92093-0949

**Keywords:** Episodic memory, fMRI, medial temporal lobe, multivoxel pattern analysis, retrieval

## Abstract

**Introduction:**

The medial temporal lobe supports integrating the “what,” “where,” and “when” of an experience into a unified memory. However, it remains unclear how representations of these contextual features are neurally encoded and distributed across medial temporal lobe subregions.

**Methods:**

This study conducted functional magnetic resonance imaging of the medial temporal lobe, while participants retrieved pair, spatial, and temporal source memories. Multivoxel classifiers were trained to distinguish between retrieval conditions before and after correction for mean signal and response times, to more thoroughly characterize the multivoxel signal associated with memory context.

**Results:**

Activity in perirhinal and parahippocampal cortex dissociated between memory for associated items and memory for their spatiotemporal context, and hippocampal activity was linked to memory for spatial context. However, perirhinal and hippocampal classifiers were, respectively, driven by effects of mean signal amplitude and task difficulty, whereas the parahippocampal classifier survived correction for these effects.

**Conclusion:**

These findings demonstrate dissociable coding mechanisms for episodic memory context across the medial temporal lobe, and further highlight a critical distinction between multivoxel representations driven by spatially distributed activity patterns, and those driven by the regional signal.

## Introduction

Episodic memories comprise multiple contextual details about a prior experience, integrating information about people or objects that were present, with their location and the temporal sequence of events that occurred. The brain's medial temporal lobe (MTL) is known to support episodic memories (Squire et al. 2004), with convergent inputs from subregions that integrate these details into a cohesive memory trace. Electrophysiology studies in rodents and nonhuman primates suggest that ensemble activity of neurons in distinct MTL subregions encode spatial, temporal, and item memory content. Furthermore, there is evidence that reactivation of the same neural subpopulations activated at encoding elicits retrieval of the original memory engram (Garner et al. 2012; Liu et al. 2012). While some of these findings have been extrapolated to humans, it is unclear whether the human MTL adopts similar means of coding contextual memories as those observed in animals.

Animal electrophysiology studies indicate that the context of an experience is represented in the coordinated activity of neurons tuned to particular features. Perhaps the best studied examples of such coding mechanisms are hippocampal place cells and entorhinal grid cells which selectively fire in preferred spatial locations of the animal's environment (Moser et al. 2008). Similarly, other studies have demonstrated hippocampal time cells that signal memory for specific moments in time, as well as stimulus-selective item cells in the perirhinal cortex (Naya and Suzuki 2011; Eichenbaum 2013). In support of an analogous neural coding method in humans, single-neuron recordings in neurosurgical patients have shown MTL activity that signals item identity (Quiroga et al. 2005), hippocampal activity signaling temporal order (Paz et al. 2010), and grid-like spiking in the entorhinal cortex (Jacobs et al. 2013).

Human neuroimaging studies are broadly consistent with the animal literature, indicating that MTL subregions integratively support item and spatiotemporal memory context (Davachi 2006; Eichenbaum et al. 2012). In particular, the perirhinal cortex has been implicated in item novelty and recognition (Davachi et al. 2003; Kohler et al. 2005; Staresina et al. 2012). Activity in the parahippocampal cortex signals memory for both temporal order (St Jacques et al. 2008; Jenkins and Ranganath 2010; Tubridy and Davachi 2011; Hseih et al. 2014) and spatial location (Ekstrom et al. 2011), and has been proposed to subserve memory for the contextual background of an experience (Bar et al. 2008). These and other studies consistently report hippocampal responses during item, spatial and temporal memory, as well as successful recollection, source retrieval or relational memory (Giovanello et al. 2004; Kohler et al. 2005; Ross and Slotnick 2008; Jenkins and Ranganath 2010; Ekstrom et al. 2011; Tubridy and Davachi 2011; Hseih et al. 2014), indicating that the hippocampus integrates multiple episodic details into a cohesive memory. While these findings together suggest that a distributed neural code in the MTL represents episodic memory context, it is unclear whether such a distributed code characterizes the type of contextual details brought up by directed source memory retrieval.

Functional magnetic resonance imaging (fMRI) studies have traditionally been used to inform about the regional involvement of brain structures in a given task, but such techniques offer little insight into additional coding mechanisms beyond aggregate regional signal magnitude. Rather than averaging signal across an area, as is typical of univariate analyses, multivariate analysis techniques can examine multivoxel activity patterns that may be sensitive to nonuniformly distributed patterns of neural activity (Mur et al. 2009; Serences and Saproo 2012). Recent applications of these methods indicate that activity patterns in the MTL may encode the content or context of a remembered experience (For review of multivoxel pattern analysis applications to episodic memory, see also Chadwick et al. 2012; Rissman and Wagner 2012). For example, studies report that voxel-wise blood oxygen level dependent (BOLD) responses in the MTL can predict subsequent memory (Watanabe et al. 2011), code subjective recognition success (Rissman et al. 2010), identify an individual's virtual location (Hassabis et al. 2009), represent distinct stimulus categories (Liang et al. 2013) or distinguish between retrieval of distinct past experiences (Chadwick et al. 2011).

Given this evidence that distributed MTL activity may support multiple levels of retrieval processing, from representing specific memory content or categorical information to predicting memory acquisition and subjective mnemonic states, it is feasible that it also characterizes the domain of a retrieved context. For instance, distinct neural ensembles might represent variants of a given feature, such as left versus right spatial location. Such populations would more highly overlap with one another than with a population coding a less similar property such as temporal order. Thus, within a region that supports spatial memory, the multivoxel activation patterns common to spatial retrieval events might be distinguishable from those during nonspatial retrieval. Indeed, in comparing activity elicited by retrieval of memories containing even distinct content, the more related the learned context, the more similar their MTL activation patterns (Hseih et al. 2014). A region's activity patterns might show greatest overlap when retrieving instances of memory features it supports, and be discriminable from more random activity patterns during retrieval of features it does not support. This study combined fMRI of the MTL with multivoxel pattern classification to test the hypothesis that spatially distributed activity patterns in MTL subregions differentially inform about the class (spatial, temporal or pair) of retrieved contextual information.

A critical assumption of multivoxel classification analyses is that class discrimination is driven by differences in the pattern of voxel-wise activity between classes of interest. However, classifier models may heavily weight additional sources of between-class differences if those signals improve classifier performance. Of particular concern is that pattern classifiers may detect between-condition differences in the global signal rather than variations in its spatial pattern. Furthermore, classifiers may be sensitive to behavioral effects that covary with the conditions of interest, such as task difficulty. Additional steps can be taken to more fully characterize the underlying signal and expose the contribution of distributed activation patterns. The contribution of global signal can be explored by examining the effects of controlling for the mean signal intensity before performing classification. The contribution of task difficulty can be explored by examining the effects of controlling for a behavioral proxy, such as response time, before performing classification (Todd et al. 2013). Thus, to account for the influence of differences between classes in the mean signal intensity or task difficulty, classifiers were trained both with and without controlling for mean signal and response times.

## Materials and Methods

### Participants

Twenty young adults were recruited from the University of California, San Diego (UCSD) and surrounding community and gave informed written consent according to UCSD Institutional Review Board requirements. All subjects were right-handed, free of psychiatric or neurological disorders and had normal to corrected vision. One session was aborted due to participant claustrophobia and two others were excluded due to excessive motion artifacts. Data from the remaining seventeen subjects (seven male, mean age ± standard deviation = 23.0 ± 3.7 years) were included for analysis.

### Stimuli and experimental paradigm

**Figure 1:**
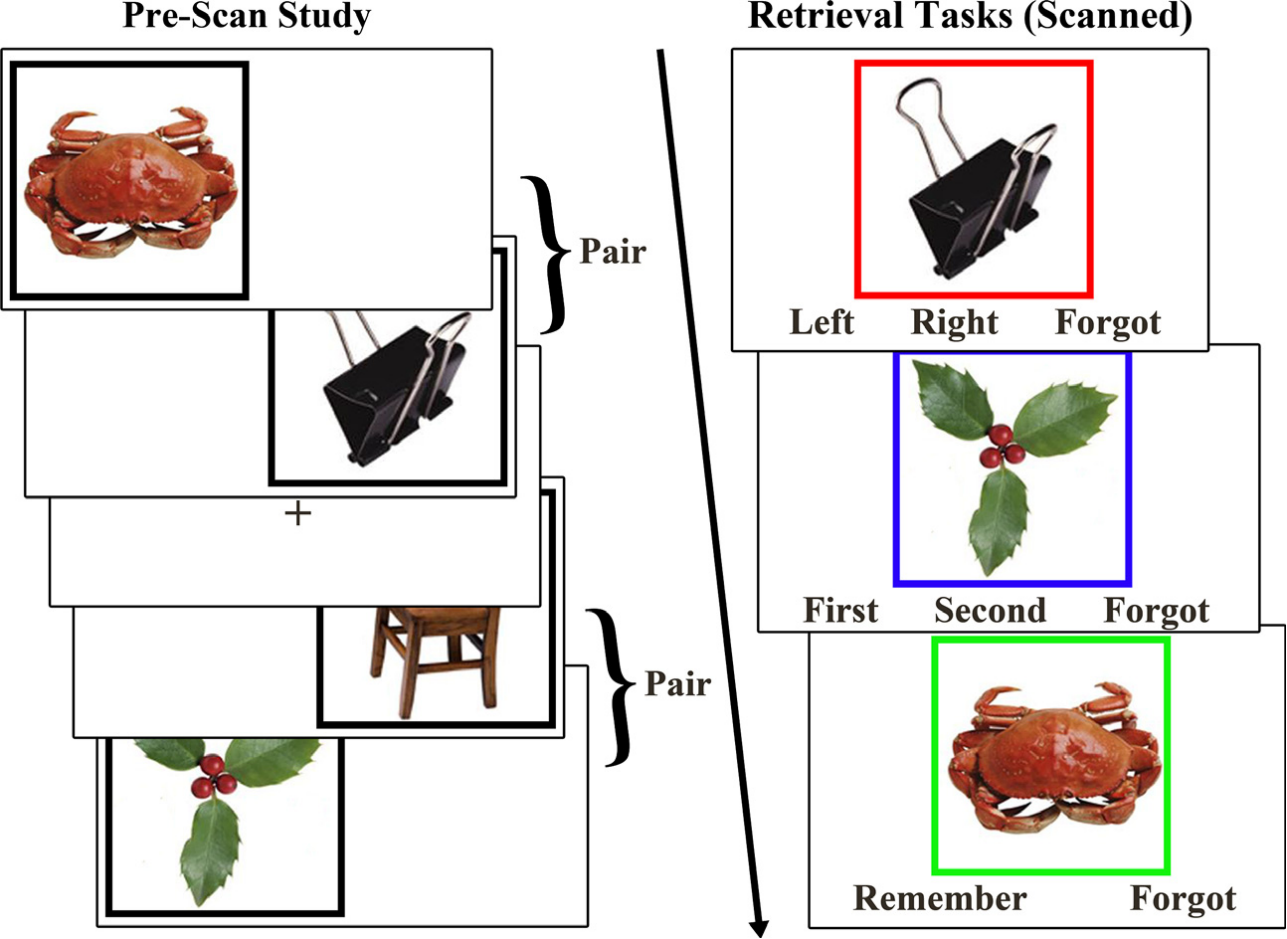
Behavioral protocol. Prior to scanning, participants studied sequentially presented object pairs, presented at either the left or right. During fMRI scanning, participants were cued with a previously studied object and performed three contextual retrieval tasks: recall the spatial location of the item (spatial, red cue), recall the temporal order of the item (temporal, blue cue), or recall the item's pair (pair, green cue).

Two hundred and forty color pictures of common objects (Bakker et al. 2008) were pseudorandomly combined into 120 pairs screened for obvious semantic associations. Prior to fMRI scanning, participants completed an associative encoding task on sequentially presented object pairs (Fig.1, left). Each object was displayed at either the left or right of a computer screen for 2-s. Objects from the same pair were separated by a 2-s blank screen and trials were separated by a 2-s fixation cross. Participants were instructed to memorize each object pair, but were given no explicit instructions to remember the location or order of the objects. Each pair was studied three times, distributed across six blocks.

Approximately 20 min after encoding, participants performed three retrieval tasks during event-related fMRI scanning (Fig.1, right). Each of the 240 items from the studied pairs was assigned to either a spatial, temporal or pair retrieval condition, and condition assignments were counterbalanced across subjects. Trials were initiated with a red, blue or green box in the center of the screen for 1-s to cue the onset of a spatial, temporal or pair retrieval trial. A previously studied object was displayed in the box for 1-s, followed by a 2-s poststimulus period. Subjects were instructed to recall the presented object from the encoding task and respond whether the object appeared at left or right or they forgot (spatial condition), first or second in the pair or they forgot (temporal condition) or to report whether they recalled or forgot the paired object (pair condition). Subjects were instructed to respond as quickly and accurately as possible with their right hand using a four-button response box. Trials were jittered with 0.5–13 s of fixation, calculated to optimize the study design for modeling the hemodynamic response to trials (Dale and Buckner 1997; Dale 1999), and to ensure that intervals following each trial were optimally balanced across conditions. Eighty trials of each condition were distributed across five 387.5-s runs.

After scanning, a cued recall test was administered to assess reliability of self-reported recall judgments from the scanned item retrieval task. One object from each pair presented in the pair condition was randomly selected as a cue, and subjects were instructed to report the associated object.

### Image data acquisition and preprocessing

**Figure 2:**
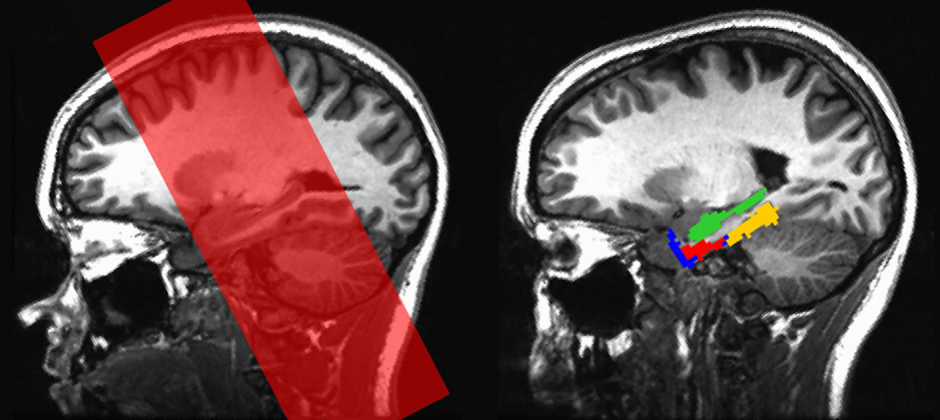
Functional imaging protocol and medial temporal lobe (MTL) regions of interest. For functional imaging, 27 slices (2 mm) were oriented perpendicular to the long axis of the hippocampus (left). Medial temporal lobe regions of interest (hippocampus, green; perirhinal cortex, blue; entorhinal cortex, red; parahippocampal cortex, yellow) were manually drawn on each subject's high-resolution structural image (right). Images show examples from single subjects.

Imaging data were acquired on a 3.0 Tesla General Electric scanner at the UCSD Keck Center for Functional MRI. Echo-planar images were collected using a gradient-echo T2*-weighted pulse sequence (2 × 2 mm in-plane resolution, 2500 ms repetition time, 30 ms echo time, 90° flip angle, 128 × 128 matrix, 256 mm field of view, 2 mm slice thickness, no gap). Each volume contained 27 slices oriented perpendicular to the long axis of the hippocampus (Fig.2, left). The first five volumes were discarded to allow for signal equilibration. ASSET calibration was performed to enable parallel imaging and field maps were acquired to correct for static field inhomogeneities (Smith et al. 2004). A high-resolution anatomical scan (1 mm × 1 mm in-plane resolution, 1.2 mm slice thickness) was collected using an inversion recovery prepared spoiled gradient recalled T1-weighted sequence.

Functional data were corrected for spatial distortions using field maps (Smith et al. 2004) and reconstructed using the AFNI suite of programs (Cox 1996). Images were slice-time corrected, corrected for motion and concatenated, and nonbrain voxels were removed using a threshold mask of the functional data.

### Univariate fMRI analysis

Prior to univariate analysis, standard landmarks were manually defined on the anatomical images, and both anatomical and functional images were normalized to Talairach space (Talairach and Tornoux 1988). The region of interest large deformation diffeomorphic metric mapping (ROI-LDDMM) alignment technique was applied to improve alignment of the MTL between subjects (Miller et al. 2005). For each subject, previously described landmarks were used to define the left and right hippocampus (Chera et al. 2009), perirhinal and entorhinal cortices (Insausti et al. 1998) and parahippocampal cortex (Stark and Okado 2003), on Talairach transformed images. These anatomical regions of interest for each subject were normalized using ROI-LDDMM to a modified model of a previously created template segmentation (Kirwan et al. 2007). Functional imaging data underwent the same ROI-LDDMM transformation as was applied to the anatomical data.

Functional runs were smoothed with a 4 mm full-width half-maximum Gaussian blur. Trials were sorted according to retrieval condition (spatial, temporal, pair) and response (correct/incorrect/forgot for spatial and temporal trials; remember/forgot for pair trials), and incorrect and forgotten trials were combined for analysis. A general linear model was constructed with regressors for each task condition (spatial correct, temporal correct, pair remember, spatial incorrect/forgot, temporal incorrect/forgot, and pair forgot) along with six motion regressors obtained from the registration process. Signal deconvolution with TENT basis functions (Cox 1996) was used to estimate the hemodynamic response for the 15 s following the stimulus onset. Beta values from 5–10 s poststimulus for correct spatial, temporal and pair conditions were extracted for each subject in the structurally defined left and right MTL regions (number of voxels per bilateral region: hippocampus, 114; perirhinal cortex, 106; entorhinal cortex, 53; parahippocampal cortex, 77). Repeated measures Analysis of Variance (ANOVA) with factors of retrieval condition, region and hemisphere was conducted to examine regional MTL activation across contextual retrieval conditions.

### Multivariate fMRI analysis

Before multivariate analyses, no smoothing or registration to standard space was performed. MTL regions of interest were drawn on each subject's anatomical image in native space (Fig.2, right). Mean (±standard deviation) number of voxels for each bilateral region were: hippocampus = 776 ± 71; perirhinal cortex = 684 ± 140; entorhinal cortex = 472 ± 63; parahippocampal cortex = 792 ± 122. Voxel counts differed by region (*F*_3,48_ = 38.84; *P *<* *0.001), related to fewer voxels for the entorhinal cortex than all other regions (*P*s < 0.001), and fewer voxels for the perirhinal cortex than the hippocampus and parahippocampal cortex (*P*s < 0.05). General linear regression was performed, with each trial modeled as a separate regressor to estimate the response amplitude to each stimulus (3dDeconvolve –stim_times_IM in AFNI; http://afni.nimh.nih.gov/pub/dist/doc/program_help/3dDeconvolve.html), and motion parameters included as regressors of no interest.

Multivoxel classification analysis was performed to identify MTL activity patterns that distinguish between spatial, temporal and pair retrieval. Analyses were conducted on each subject using the LIBLINEAR support vector machine (SVM) package (http://www.csie.ntu.edu.tw/~cjlin/liblinear/) implemented in a custom Matlab script. Features were voxel-wise signal estimates from the third (5 s) or fourth (7.5 s) scans. These time-points were selected to capture the peak hemodynamic response, which may vary across brain regions and is estimated to occur at a 6–8 s delay (Friston et al. 1994). Feature examples included correct retrieval trials, coded according to spatial, temporal or pair condition. Binary classifiers were trained and tested on anatomically defined regions of interest, including left and right hippocampus, perirhinal cortex, entorhinal cortex and parahippocampal cortex, to distinguish spatial from nonspatial, temporal from nontemporal, and pair from nonpair retrieval conditions. Binary classifiers were selected to isolate activity patterns selectively associated with a single condition, in contrast to a three-way classifier which may indicate a difference between conditions, but would carry ambiguous information about the most informative class. Trials from the larger class were randomly down-sampled prior to training to ensure equal trial numbers in each training class.

Eighty percent of trials were allocated to training, and the remaining twenty percent reserved for testing. Within the 80% of data allocated for training, five-fold cross-validation, divided along run boundaries, was performed to determine the optimal regularization parameter C from a range of 10^−10^ to 1. This C value was used to train a classifier model on the full training dataset which was then tested on the independent test dataset.

Group-level classifier accuracy was computed using a one-sampled t-test versus chance (50%) and assessed for significance with permutation testing. For permutation testing, class trial labels were randomized and the training and testing were conducted as described above. For each of 3000 permutations, a t-value was computed from classification accuracies across all subjects versus chance. The real t-value was compared to the permutation distribution of t-values, and t-values in the top 5% were considered significant.

Classifier accuracy may be sensitive to between-class differences in the mean signal amplitude or levels of task difficulty. To further characterize the bases of the classifiers, voxel-wise projection matrices were constructed to remove (1) the mean signal amplitude, and (2) signal modulation by response time. Before classifier training, the voxel-by-trial matrix of response amplitudes (D) was corrected for the mean signal or response times as follows, where P = the relevant projection matrix: D' = D − PD. For mean correction, the projection matrix P = m * m', where m = a vector of voxel-wise mean responses. For response time correction, the projection matrix P = E − (r * inv(r' * r) * r'), where E = the identity matrix of D and r = a vector of trial response times. (Code available upon request).

## Results

### Behavioral performance

Participants correctly recalled 84 ± 3% (mean ± standard error) of both spatial and temporal retrieval trials and reported remembering 79 ± 4% of pair trials. They reported forgetting 10 ± 2% of spatial, 6 ± 2% of temporal and 16 ± 4% of pair retrieval trials. 88 ± 4% of pairs reported remembered during scanning were correctly recalled during the postscan cued recall test, suggesting that participants' self-reported memory judgments were reliable.

Mean response times to correct spatial, correct temporal, and remembered pair trials were, respectively, 1284 ± 76, 1384 ± 74 and 1183 ± 81 msec. Response times differed between conditions (*F*_2,32_ = 17.41, *P *<* *0.001). Pairwise comparisons indicated longer response times for temporal than spatial (*P *<* *0.01) or pair (*P *<* *0.001) and for spatial than pair (*P *<* *0.01) trials.

### Univariate fMRI

**Figure 3:**
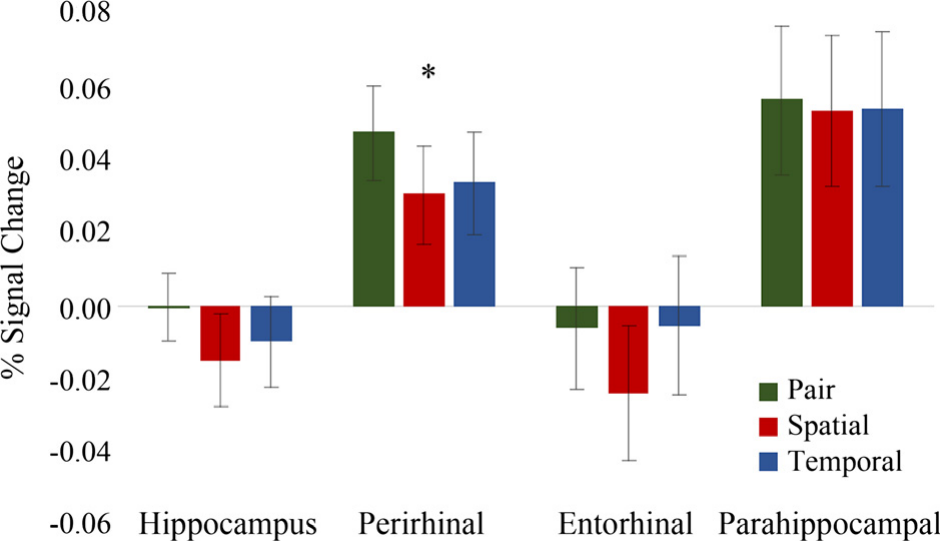
Medial temporal lobe (MTL) activity during pair, spatial and temporal retrieval. Percent signal change during the pair, spatial and temporal retrieval conditions are shown for the bilateral hippocampus, perirhinal cortex, entorhinal cortex and parahippocampal cortex. Activity in the perirhinal cortex was greater during pair than spatial or temporal retrieval. *P <* *0.05, corrected for multiple comparisons.

Group-level univariate analysis was used to examine MTL activity selectively associated with spatial, temporal or pair retrieval. A main effect of MTL region (*F*_3,48_ = 7.11, *P *<* *0.001), as well as a region by hemisphere interaction (*F*_3,48_ = 4.64, *P *<* *0.01) and a region by hemisphere by retrieval condition interaction (*F*_6,96_ = 2.67, *P *<* *0.05), were observed. To further probe regional effects, activity within each MTL region was examined separately (Fig.3, Table 1). Activity in the perirhinal cortex differed by retrieval condition (*F*_2,32_ = 4.06, *P *<* *0.05), with greater activity for pair than spatial or temporal retrieval (*P*s < 0.05). A trend for a difference between retrieval conditions occurred in the hippocampus (*F*_2,32_ = 2.78, *P *=* *0.08), related to greater activity for pair than spatial retrieval (*P *<* *0.05), and in the entorhinal cortex (*F*_2,32_ = 2.57, *P *=* *0.09), related to greater activity during temporal than spatial retrieval (*P *<* *0.05). Although an interaction between retrieval condition and hemisphere occurred in the parahippocampal cortex (*F*_2,32_ = 7.83, *P *<* *0.01), activity did not differ between conditions within left (*P *=* *0.11) or right (*P *=* *0.34) parahippocampal cortex. Notably, the perirhinal and parahippocampal cortices were positively activated in all task conditions, whereas the hippocampus and entorhinal cortex were negatively activated below baseline.

**Table 1:** *F*-values for activation differences between retrieval conditions in each medial temporal lobe (MTL) subregion

	Hippocampus	Perirhinal**	Entorhinal	Parahippocampal
Volume (mm^3^)	7296	6784	3392	4928
Pair versus Spatial (*F*-value)	4.75*	4.62*	2.79	0.09
Pair versus Temporal (*F*-value)	2.01	6.42*	0.01	0.06
Spatial versus Temporal (*F*-value)	0.92	70.36	6.93*	0.01

** Significant effect of condition (*P *<* *0.05, corrected for multiple comparisons).

* Significant pairwise difference (*P *<* *0.05).

### Multivariate fMRI

Subject-specific SVM classifiers were trained on voxel-wise activity patterns in left and right MTL subregions to distinguish spatial from nonspatial, temporal from nontemporal and pair from nonpair retrieval. Classification accuracies were assessed for significance with random permutation testing. Classifiers were first trained without accounting for the mean signal intensity or response times. The hemodynamic response peaked 5 s poststimulus for the parahippocampal cortex, and at 7.5 s for the hippocampus, entorhinal cortex and perirhinal cortex. Therefore, classifiers were tested at both 5 and 7.5 s poststimulus for each region. Any classifiers that performed significantly above chance when trained and tested on the untransformed data were retrained 1) after projecting out the mean signal amplitude, to evaluate classifier performance attributable to the spatial distribution of activity patterns, uncontaminated by between-class differences in signal magnitude, and 2) after controlling for the effect of response times on signal magnitude, to evaluate classifier performance independent of task difficulty.

**Figure 4:**
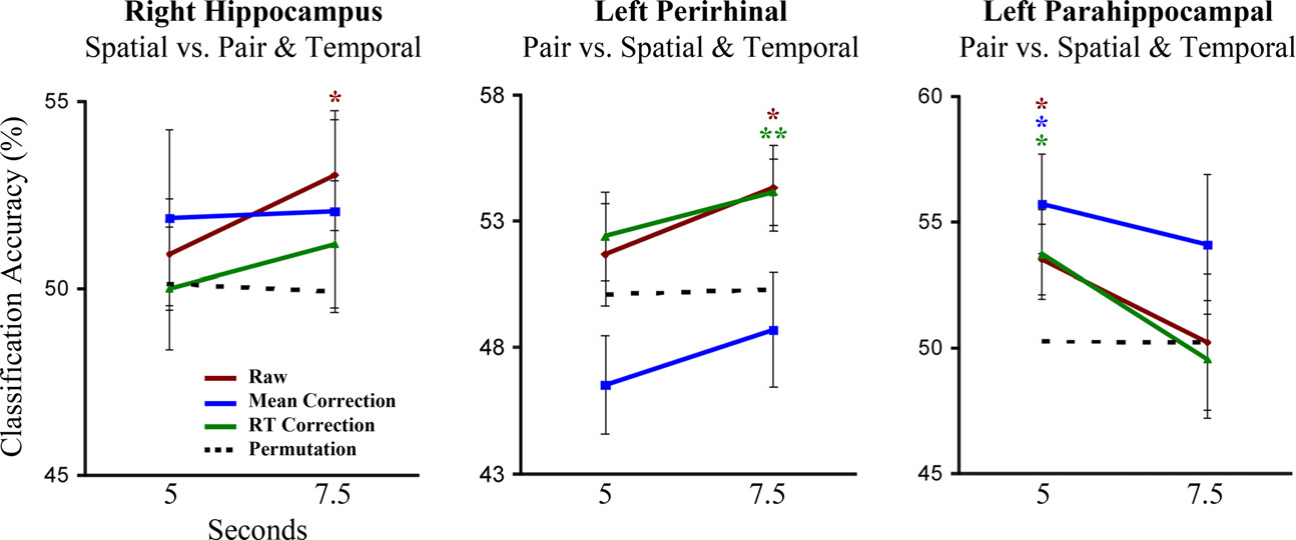
Medial temporal lobe (MTL) multivoxel classification accuracy. Support vector machine (SVM) classifiers were trained on untransformed multivoxel signal estimates (red), after projecting out the mean signal amplitude (blue) or after controlling for response times (green) and were tested for significance relative to permutation testing (dotted). Multivoxel activity in the right hippocampus at 7.5 s distinguished spatial from nonspatial retrieval above chance when trained on raw signal, but not after controlling for the mean signal or response times (left). Left perirhinal cortex activity at 7.5 s distinguished pair from nonpair retrieval using raw signal and after controlling for response times, but not after controlling for the mean signal (middle). Left parahippocampal cortex activity at 5 s classified pair versus nonpair retrieval using raw signal and after controlling for the mean signal and response times (right). Error bars represent the standard error of the mean. *P <* *0.05, **P <* *0.01.

Classifiers trained on multivoxel activity in the right hippocampus at 7.5 s poststimulus distinguished spatial from temporal and pair trials (mean classification accuracy = 53.0 ± 1.5%; *t*_16_ = 2.03, one-sampled t-test vs. 50%; *P *<* *0.05, vs. permutation). However, this effect did not survive Bonferroni correction for multiple comparisons across hemispheres. Furthermore, classifier accuracy was no longer significant after mean projection (52.1 ± 2.7%; *t*_16_ = 0.77; *P *=* *0.57) or after controlling for response times (51.2 ± 1.7%; *t*_16_ = 0.69; *P *=* *0.25). Spatial memory classifiers trained on hippocampal activity at 5 s did not differ from chance (raw: *P *=* *0.30; mean correction: *P *=* *0.59; response time correction: *P *=* *0.56) (Fig.4, left).

Classifiers trained on left perirhinal cortex activity patterns at 7.5 s distinguished pair retrieval from spatial and temporal retrieval (accuracy = 54.3 ± 1.7%; *t*_16_ = 2.54; *P *<* *0.05). Classification accuracy remained significant after controlling for response times (54.1 ± 1.3%; *t*_16_ = 3.12; *P *<* *0.01), but did not differ from chance after mean projection (48.7 ± 2.3%; *t*_16_ = −0.57; *P *=* *0.86). The accuracies of classifiers trained on uncorrected signal and after correction for response times survived correction for multiple comparisons across hemispheres. Perirhinal cortex classifiers trained to distinguish pair retrieval at the 5 s time-point did not differ from chance (raw: *P *=* *0.23; mean correction: *P *=* *0.99; response time correction: *P *=* *0.11) (Fig.4, middle).

Classifiers trained on left parahippocampal activity patterns at 5 s distinguished pair retrieval from spatial and temporal retrieval (accuracy = 53.5 ± 1.4%; *t*_16_ = 2.52; *P *<* *0.05). Classifiers remained accurate after controlling for the mean signal (55.7 ± 2.0%; *t*_16_ = 2.88; *P *<* *0.05) and response times (53.7 ± 1.8%; *t*_16_ = 2.11; *P *<* *0.05). (Fig.4, right) However, only the accuracy of the classifier trained on uncorrected signal survived correction for multiple comparisons across hemispheres. Pair retrieval classifiers trained on parahippocampal cortex activity at 7.5 s did not differ from chance (raw: *P *=* *0.51; mean correction: *P *=* *0.26; response time correction: *P *=* *0.63).

Classifiers trained on entorhinal cortex activity were unable to distinguish between retrieval conditions. Due to the lower number of voxels in the entorhinal cortex relative to the other MTL regions (*P *<* *0.001) and the lack of effects in this area, the entorhinal cortex was excluded from subsequent analyses.

To compare classifier accuracy across regions, a region by hemisphere by time-point ANOVA was conducted for each contrast. Accuracies for the *spatial versus pair and temporal* classifier differed by region (*F*_2,32_ = 7.15, *P *<* *0.01). Paired comparisons indicated higher accuracy for the hippocampus than perirhinal and parahippocampal cortices (*P*s < 0.01). Accuracies for the *pair versus spatial and temporal* classifier did not differ across regions (*P *=* *0.59).

It is important to note that multiple classifiers were tested to examine differences associated with temporal or spatial properties of the BOLD signal. Even with reducing comparisons by focusing on the MTL, classifier significance would not have survived correction for the 48 comparisons tested here (three contrasts, four regions, two hemispheres, two time-points); thus, some caution should be taken in interpreting these results. Nonetheless, these findings align with existing models of MTL subregion function, and serve as a hypothesis-generating foundation upon which to build with targeted, replication-based follow-up investigations.

## Discussion

This study used univariate and multivariate analyses to examine both global engagement of MTL subregions and their spatially distributed activity patterns during contextual memory retrieval. Spatial and pair memory signals were present in both the aggregate across-voxel response and multivoxel patterns. Critically, these SVM classifiers were sensitive to between-class differences in mean signal and modulation by response times, indicating that not all multivoxel effects are directly related to differences in fine-scale activity patterns.

### Response-time-dependent hippocampal activity distinguishes spatial from nonspatial retrieval

A pattern classifier trained on BOLD signal in the right hippocampus effectively distinguished spatial from nonspatial memory retrieval. However, follow-up analyses revealed that classification accuracy was diminished when accounting for either the mean signal or trial-by-trial response times. Thus, discriminability of the hippocampus was more likely attributable to a large-scale regional response that was not detected at the group-level, rather than a small-scale spatial activity pattern specific to spatial retrieval.

The discriminating hippocampal signal was related to response times, suggesting that the between-condition signal difference co-varies with task difficulty. This finding is consistent with evidence that some hippocampal activity during retrieval can be regulated by retrieval effort (for review, see Reas and Brewer 2013b), as it is reduced during the retrieval of lower strength memories and negatively correlates with retrieval response times (Reas et al. 2011; Reas and Brewer 2013a). Based on these findings, a difference in the mean signal between spatial and nonspatial retrieval might be expected from a univariate contrast. Interestingly, this analysis showed a trend for reduced hippocampal activity during spatial retrieval relative to pair retrieval. This discrepancy highlights a critical distinction between univariate group-level and multivariate subject-level approaches. In particular, multivoxel approaches are especially sensitive to spatially distributed information, versus global activation, and are less sensitive to subject-level variability than univariate methods (Jimura and Poldrack 2012; Davis et al. 2014). Here, differences between the univariate and multivariate findings underscore their distinct sensitivities to group-level and subject-level effects. Whereas the former is exclusively sensitive to effects in which the directionality is consistent across individuals, the latter is sensitive to any difference between test conditions, regardless of their directionality. Therefore, if spatial retrieval is inconsistently more or less difficult than nonspatial retrieval across participants, and task difficulty strongly modulates the hippocampus, an effect on hippocampal activity would emerge at the subject level (i.e. multivoxel classification) but not at the group level (i.e. univariate analysis).

Consistent with our findings, prior studies have reported hippocampal involvement in spatial location source memory for studied items (Ross and Slotnick 2008). It is well established that spatial representations in animals are carried, at least in part, by a population-level neural code in the hippocampus (Moser et al. 2008), and emerging evidence supports a spatially distributed neural code for spatial location in humans (Hassabis et al. 2009). However, this study failed to find evidence for a multivoxel code unique to spatial, versus other contextual memory retrieval, beyond that driven by response times. Spatial memory encompasses memory for the location of experiences, objects in our external environment and our personal sense of position in relation to that environment, distinct functions subserved by different brain networks (Suzuki et al. 2005). Animal studies have shown that the hippocampus is essential for binding spatial, temporal and object details into memory (Ergorul and Eichenbaum 2004), and that hippocampal neurons integratively code (Kraus et al. 2013), and similarly pattern separate (Azab et al. 2013), temporal and spatial information, which may suggest a patterned representation in the hippocampus that generalizes across contextual domains and where differences could be more related to response time than to particular contexts. Despite prior evidence that the hippocampus is involved in associative or relational memory (Sperling et al. 2003; Davachi 2006), the multivoxel signal did not distinguish pair from spatiotemporal memory. If information about associated objects and their spatiotemporal context are highly integrated by the hippocampus, the neural representations of a recalled object and its context might overlap, and thus be indistinguishable.

### The perirhinal cortex distinguishes pair from spatiotemporal retrieval

Univariate fMRI analyses revealed bilateral activation of the perirhinal cortex during pair compared to spatiotemporal retrieval. Multivariate analyses extended this finding to suggest that multivoxel activity patterns in the left perirhinal cortex additionally distinguished between pair and nonpair retrieval conditions. Although the multivoxel classifier was robust to the influence of response time, its performance was largely driven by the mean BOLD signal. This is consistent with prior research showing that mean activity in the perirhinal cortex signals memory for items, unified concepts, or object familiarity (Davachi et al. 2003; Kohler et al. 2005; Davachi 2006; Mayes et al. 2007; Staresina et al. 2012). Thus, while these findings support the regional involvement of the perirhinal cortex in object retrieval that is independent of task difficulty, they provide no evidence for a spatially distributed neural code selective for the process of item retrieval. Rather, the perirhinal cortex may be globally engaged while retrieving item information, with distinct neural representations for object identity (Naya and Suzuki 2011; Hseih et al. 2014).

### Parahippocampal cortex activity patterns distinguish pair from spatiotemporal retrieval

Activity patterns in the left parahippocampal cortex also effectively discriminated between pair and spatiotemporal retrieval. However, in contrast to the perirhinal cortex classifier, the parahippocampal classifier was robust to effects of both the mean signal and response times. Notably, the univariate analysis did not demonstrate differences in parahippocampal activity between retrieval conditions. These findings suggest that the spatial distribution of parahippocampal cortex activity discriminates between an item and spatiotemporal content associated with a retrieved memory, even when this signal is absent from the global response.

Although multivoxel classifiers can identify discriminative activity pattern differences, they do not inform about which class carries the more discriminating representation. Thus, in this comparison (pair vs. nonpair), the multivoxel patterns may code for either aspect of a recalled memory's context, either its associated item or its integrated spatiotemporal features. Support for both possibilities exists. Prior research has demonstrated parahippocampal involvement in both spatial and temporal memory context (St Jacques et al. 2008; Jenkins and Ranganath 2010; Ekstrom et al. 2011; Mullally and Maguire 2011; Tubridy and Davachi 2011) and activity patterns that distinguish between spatial environments (Hassabis et al. 2009). Yet, parahippocampal activity patterns were also reported to differentiate visual stimulus classes (Diana et al. 2008), together suggesting that such patterns could encode either spatiotemporal context or categorical object information. More broadly, the parahippocampal cortex is thought to subserve contextual associations (Bar et al. 2008), and could thus differentially represent the item and spatiotemporal associations in this study. Such functional diversity may be accounted for by anatomically heterogeneity within the parahippocampal cortex, as distinct connectivity and cytoarchitectonic profiles have been identified in subregions of both the monkey (TH, TF) and human parahippocampal cortex (Suzuki and Amaral 1994, 2003; Baldassano et al. 2013). Although additional research is needed to disambiguate which precise contextual features are represented by parahippocampal activity patterns, these findings provide compelling evidence for a distributed code for episodic memory context in the parahippocampal cortex.

### Limitations

It is important to note that even for those classifiers with above chance performance, the absolute accuracy values were relatively low. Furthermore, not all significant classifications survived correction for multiple comparisons across hemispheres; while classifiers trained on raw signal from the perirhinal and parahippocampal cortices survived correction, the classifier trained on signal from the hippocampus did not. Follow-up studies will therefore be important to determine the reliability of this signal. While we acknowledge the possibility of false positives, there are several reasons why a weaker signal in this dataset may have reduced classifier discriminability. Compared to BOLD signal in other brain regions, that in the MTL is reduced and is particularly vulnerable to susceptibility artifacts (Cohen and Bookheimer 1994; Olman et al. 2009). Furthermore, classification accuracy is highly variable across studies and depends heavily on the complexity of represented information (Chadwick et al. 2012). Here, the paradigm was designed to elicit recall of distinct features of a complex memory, yet the associative nature of these memories may have promoted overlap in retrieved details (e.g., some spatial information retrieved on a temporal trial). Thus, while each condition should more heavily weigh the target condition than the two nontarget conditions, there may be substantial overlap in the retrieval processes evoked across conditions.

Additionally, the decision to use classifiers comparing one condition against the other two conditions, as opposed to comparing each condition against every other condition, was driven by an interest in isolating distinct features of the target condition tested against the backdrop of a more heterogeneous sample. Possible limitations of this approach are that, in each test, one of the two combined conditions could more strongly influence classifier performance than the other. Nevertheless, developing separate classifier models, which each isolated one condition from the others, allowed a more efficient analysis of whether a particular brain region held a distinguishable pattern for a selected target feature of retrieval. This approach was therefore chosen, as it was most suited to examine the posed hypotheses.

Finally, caution should be taken in attributing these findings to representations of memory content rather than retrieval orientation. Although we minimized the influence of this confound by excluding trials in which a retrieval attempt was made but was unsuccessful, future studies will be necessary to dissociate these integrated processes.

## Conclusions

Multivoxel classifiers trained on untransformed BOLD signal estimates identified three MTL subregions in which activity patterns accurately discriminated retrieval conditions. However, follow-up analyses controlling for effects related to the mean signal intensity and task difficulty revealed that only one of the three classifiers was robust against these confounds. These findings underscore the importance of fully characterizing the signal driving multivoxel effects. Future studies should incorporate appropriate controls to dissociate information represented by regional engagement from that carried in a distributed code, which may reflect meaningfully different neural coding mechanisms.
